# Effects of the “plate model” as part of dietary intervention on modification of selected cardiometabolic risk factors in post-myocardial infarction patients: study protocol for a randomized controlled trial

**DOI:** 10.1186/s13063-017-2057-6

**Published:** 2017-07-10

**Authors:** Ranil Jayawardena, Pasindu Fernando, Niroshan Lokunarangoda, Anidu Keerthi Pathirana

**Affiliations:** 1 Department of Physiology, Faculty of Medicine, University of Colombo, Colombo, Sri Lanka; 2 Institute of Health and Biomedical Innovation, Queensland University of Technology, Brisbane, QLD Australia; 3 Institute of Cardiology, National Hospital of Sri Lanka, Colombo, Sri Lanka; 4 Department of Medicine, Faculty of Medicine and Allied Sciences, Rajarata University of Sri Lanka, Mihintale, Sri Lanka

**Keywords:** Cardiovascular disease, Cardiac rehabilitation diet, Nutrition, Plate model, Sri Lanka

## Abstract

**Background:**

Cardiovascular disease remains the leading cause of morbidity and mortality worldwide, and there is a rising global burden. The effects of diet on cardiometabolic risk factors have been studied extensively. Healthy eating as a cost-effective approach to risk reduction in post-myocardial infarction patients is proven to be beneficial, and the “plate model” is one of the practical methods to achieve this objective.

**Methods/design:**

The study will be conducted as a randomized, single-blind, controlled clinical trial for a period of 3 months. A total of 120 overweight (body mass index >23 kg/m^2^) inpatients (aged 20–70 years) with a history of troponin-positive acute coronary syndrome (ACS) within the 1 month preceding the study will be recruited. Simple randomization will be used in participant allocation. The intervention group will receive the model plate diet. The control group will be provided with routine dietary advice. Other domains, such as advice on exercise and lifestyle modification, will be equalized among patients in both the groups. The visits and evaluations will be done at recruitment (visit 0), 4 weeks, and 12 weeks after the intervention. The primary outcome will be a mean body weight reduction of 10%, and the secondary outcomes will include mean reduction of systolic and diastolic blood pressure, improvement of anthropometric parameters, and improvement of lipid profile and liver enzymes in the test group compared with the control group at 12 weeks following the plate model diet.

**Discussion:**

This study protocol is designed to establish the effects of the plate model diet on modification of cardiometabolic risk factors in patients with ACS. This will also be a pioneering study designed to investigate the practicality of the model plate in local settings and in the South Asian region.

**Trial registration:**

Sri Lanka Clinical Trials Registry identifier: SLCTR/2016/22. Registered on 22 September 2016 (http://www.slctr.lk/trials/483).

**Electronic supplementary material:**

The online version of this article (doi:10.1186/s13063-017-2057-6) contains supplementary material, which is available to authorized users.

## Background

The World Health Organization (WHO) estimates that cardiovascular disease (CVD) is the leading cause of death globally [1]. Current data on the global impact of CVD show that the highest mortality due to CVD will occur predominantly in developing countries in the next decade [2]. An estimated 17.5 million people died as a result of CVD in 2012, representing 31% of all global deaths. Of these deaths, an estimated 7.6 million were due to coronary heart disease. More than three-fourths of CVD-related deaths occur in low- and middle-income countries [3]. According to another systematic review, the global burden of ischemic heart disease (IHD) increased by 29 million disability-adjusted life-years (a 29% increase) between 1990 and 2010 [4].

There are two approaches to the prevention of CVD, and they are population-wide and individual [1]. The expected goals of CVD morbidity and mortality reduction can be accomplished only through a combination of both approaches. Although large-scale studies have shown effective strategies to prevent CVD-related deaths in the past decade, most of the evidence was accumulated from the developed regions of the world. Hence, there is a need to conduct studies in different geographic regions, diverse cultures, and various ethnicities to address the paucity of high-quality data in developing countries. The INTERHEART study is a good example of a study with broader geographic and ethnic representation. INTERHEART, representing every inhabited continent, has provided a multitude of relevant data on a global scale [5]. The INTERHEART researchers have studied the association of a selection of nine risk factors for myocardial infarction (MI) and their population attributable risk. Their study showed that abnormal lipid levels, smoking, hypertension, diabetes, abdominal obesity, psychosocial factors, alcohol use, reduced consumption of fruits and vegetables, and lack of regular physical activity account for most of the risk of MI worldwide in both sexes and in all age groups in all regions [6].

As a “best buyer,” the WHO recommends healthy eating to be one of the most cost-effective preventive strategies on an individual basis [1]. Reduction of salt and fat in the diet and consumption of fruits and vegetables have long been known to yield protective effects on the heart for both primary and secondary prevention of CVD. The investigators in the Lyon heart study examined 605 patients aged 55–80 years with previous MI and found that the benefits of the Mediterranean diet could be extended to the secondary prevention of CVD. Those who followed the Mediterranean diet had a 50% to 70% lower risk of recurrent heart disease compared with those who followed a diet similar to the American Heart Association (AHA) Step-1 diet [7]. The combination of diet and statin treatment in preventing cardiovascular events was studied by Pitsavos et al. [8], and they observed a synergistic effect of a combination of the Mediterranean diet with statin treatment on coronary risk. In particular, they observed that the aforementioned combination was associated with a 43% reduction in coronary risk, independent of cholesterol levels and other cardiovascular factors [8]. According to a hospital-based case-control study conducted in India, a significant dose-dependent inverse association between vegetable intake and IHD risk exists. The inverse association was stronger for green leafy vegetables, and persons consuming a median of 3.5 servings of green leafy vegetables per week had a 67% lower relative risk than did those consuming 0.5 serving per week [9].

Dietary patterns in Sri Lanka are changing at a faster pace than ever because of rapidly evolving urbanization. Consequently, the last 2 decades have seen a threefold increase in the level of obesity among Sri Lankan adults [10]. In 2010, it was reported that the prevalence rates of overweight, obese, and centrally obese people in Sri Lanka were 25.2%, 9.2%, and 26.2%, respectively [11]. Obesity-associated metabolic diseases also have reached epidemic proportions in Sri Lanka. The prevalence of metabolic syndrome is 25% [12], hypertension approximately 20% [13], and dysglycemia 21% [14]. These comorbid factors invariably increase health care expenditures of obese persons [15]. In 2014, noncommunicable diseases accounted for 75% of the total deaths in Sri Lanka, of which CVD-related deaths alone accounted for 40% [16]. According to a study conducted in Sri Lanka, a substantial proportion of the adult population fails to follow recommended dietary guidelines [17]. Almost 70% of those studied exceeded the recommendations for starch intake; in contrast, the daily intake of fruit (0.43 servings/day) and vegetable portions (1.73 servings/day) fell well below national recommendations, with only 3.5% of adults consuming the recommended five portions of fruits and vegetables per day [17]. Thus, Sri Lankans consume over 70% of their calories as carbohydrates (mainly refined), whereas only 10% of calories are derived from protein [18]. This is not surprising, because rice is the main staple food in Sri Lanka [19]. Hence, cutting down the amount of rice per meal might be a solution to the problem of excessive intake of refined carbohydrates and energy.

Using a “model plate” is one of the practical methods to reduce the average portion size of staple food in main meals, which also could ensure the sufficient intake of vegetables and protein foods simultaneously. Food and nutrition experts recommend the “plate model” to control the rice portion and to replace it with nonstarch vegetables [20]. The local [21] and international [22] expert evidence supports the plate model for the promotion of healthy eating, whose aim is at dietary calorie reduction and redistribution of macronutrients in a healthier manner. By devising the plate model, one can ensure an increased intake of complex carbohydrates from dark green leafy salads and nonstarchy vegetables as well as high fiber from whole grain, thus lowering the glycemic load in food. Dietary fiber intake reduces chronic inflammation [23], which is one of the postulated pathophysiologies of atherosclerosis [24]. Increased consumption of vegetables ensures the high intake of beta-carotene, vitamins C and E, polyphenols, and minerals, which are vital in sustaining bodily functions. Furthermore, increased intake of plant sterols and stanols decreases the absorption of cholesterol and thereby reduces serum cholesterol levels [25], particularly low-density lipoprotein (LDL) cholesterol [26].

Although evidence exists to guide the diet of patients in post-MI rehabilitation, the lack of standard guidelines in Sri Lanka has led clinicians to commence their patients on various untested dietary schedules. Therefore, scientific evaluation of the effects of a balanced diet with the use of a plate model and its impact on coronary risk factor modification needs to be assessed locally as well as globally. In the present study protocol, we are trying to depict the method of one such study to evaluate the effects of a plate model diet on modification of selected cardiometabolic risk factors in post-MI patients.

## Methods/design

### Objectives and hypothesis

The aim of the present study is to evaluate the effects of a plate model on modification of selected cardiometabolic risk factors in patients with a history of MI. Our hypothesis is that intake of a healthy diet using the model plate will improve coronary risk factors with an expected mean weight reduction by 10% in the test group after adopting the plate model diet for a period of 12 weeks and reduction of total cholesterol, LDL cholesterol, total cholesterol/high-density lipoprotein (HDL) ratio, blood pressure (BP), and alanine aminotransferase (ALT) compared with the control group.

### Study design

**Fig. 1:**
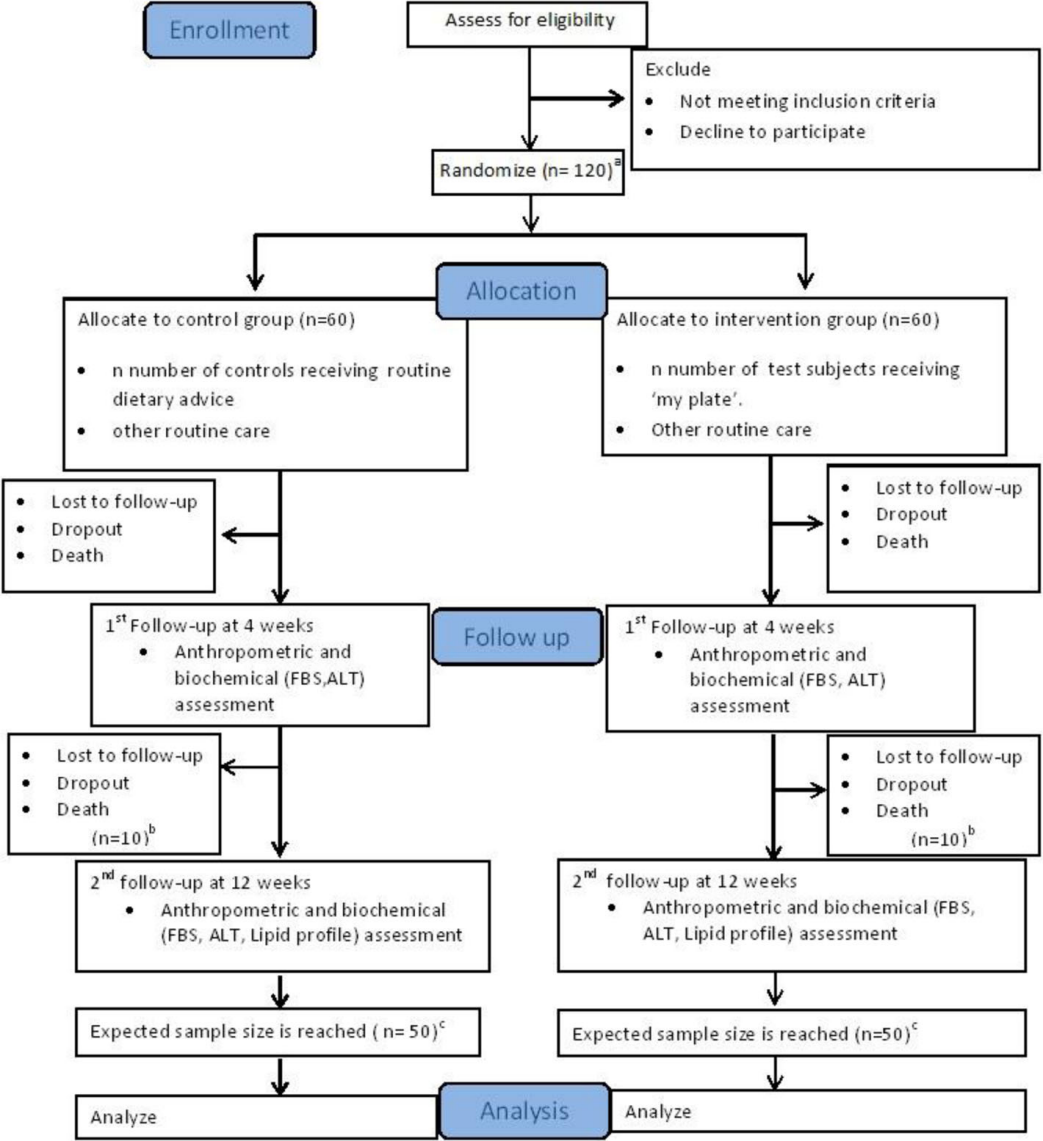
Schematic representation of the study protocol. *ALT* Alanine aminotransferase, *FBS* Fasting blood sugar

**Fig. 2:**
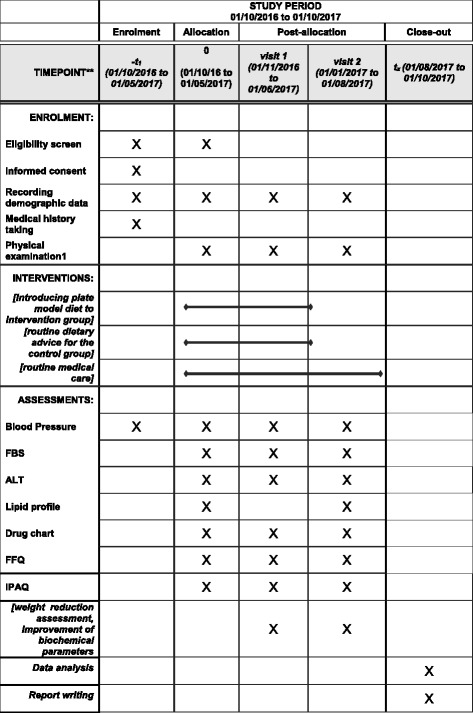
The schedule of enrollment, interventions, and assessments

This proposed study is an interventional, randomized, single-blind study with parallel subject allocation of patients with a history of troponin-positive acute coronary syndrome (ACS). A summary of the study protocol is presented in Fig. 1. The Standard Protocol Items: Recommendations for Interventional Trials (SPIRIT) checklist is presented in Fig. 2 and Additional file 1.

### Sample size

The sample size will be selected on the basis of a pilot survey, and the number needed for recruitment will be determined to account for a confidence level of 95%, a confidence interval of 10%, and 80% statistical power. A 10% hypothesized difference of participants’ body weight between the control and intervention groups following the plate model diet at the end of 12 weeks is taken as the primary outcome when calculating the sample size. Because the other biochemical parameters (BP, lipid profile, fasting blood sugar [FBS], and ALT) are prone to vary with medication and compliance with medication, they were not regarded in sample size calculations. A subsequent 5% withdrawal rate and another 15% loss to follow-up (total of 20%) are accounted for in the calculation. A total sample of 120 will be recruited over an anticipated period of 3 months.

### Study setting

The study is being conducted at the Institute of Cardiology, National Hospital of Sri Lanka, which is a tertiary care facility for patients with cardiac disease.

### Study population

The subjects will be voluntary participants admitted to the cardiology unit at the National Hospital of Sri Lanka and diagnosed with troponin-positive ACS. Subjects who fulfill the inclusion and not the exclusion criteria will be selected for the study. Patients with troponin-positive MI (i.e., ST-elevation MI or non-ST-elevation MI) or patients who fulfill the electrocardiogram (ECG) criteria as defined by the World Heart Federation Task Force for the Universal Definition of Myocardial Infarction [27] will be recruited to take part in the study, regardless of the troponin quantitative assay. Furthermore, new or presumably new left bundle branch block and true posterior MI have been considered as an ST-elevation myocardial infarction (STEMI) equivalent. Study participants will be recruited into the study after their written informed consent is obtained.

### Inclusion criteria

Post-MI patients admitted to the tertiary care facility who fulfill all of the following criteria will be selected for the study:

1. Aged between 20 to 70 years
2. Patients with a troponin-positive ACS during the 1 month preceding the date of recruitment, regardless of history of unstable angina
o STEMI defined as new ST elevation at the J-point in at least two contiguous leads of ≥2 mm (0.2 mV) in men or ≥1.5 mm (0.15 mV) in women in leads V2–V3 and/or of ≥1 mm (0.1 mV) in other contiguous chest leads or the limb leads, as well as new or presumably new left bundle branch block
o Non-ST-elevation MI is diagnosed in the absence of the aforementioned ECG changes, although in the presence of cardiac biomarker positivity (according to American College of Cardiology/AHA guidelines) cutoffs for troponin I >0.5 ng/ml and creatine kinase 5.0 ng/ml are used. The diagnosis is made by the admitting medical officer, and before recruiting the patient, the investigator will also confirm the presence of the above-described criteria.
3. Patients who have had the index coronary event that led to the current hospital admission within the preceding 1-month period
4. Patients who consume rice as a staple food for at least two main meals per day
5. Patients who consent and are able to attend clinic follow-up visits on a monthly basis
6. Patients who are eating homemade food for at least two main meals per day
7. Patients whose body mass index (BMI) is greater than 23 kg/m^2^

### Exclusion criteria

Patients who fulfill the following criteria will be excluded from the study:

1. Patients who are in end-stage renal failure (stage III or higher), have congestive cardiac failure (New York Heart Association class III and above), have chronic liver cell disease (Child-Pugh class B or above), have severe anemia (hemoglobin <8.0 g/dl for both men and women), or have other severe systemic disease at the time of recruitment
2. Patients who are pregnant or breastfeeding.
3. Patients who have had any documented infection with systemic effects (i.e., high-grade fever, abnormal leukocyte count >11,000/μl, or erythrocyte sedimentation rate >22 mm/first hour for men and 29 mm/first hour for women) within the last 2 weeks
4. Patients who are already on a modified diet or taking dietary supplements

### Suspension criteria

Patients may be withdrawn from the study in the following circumstances:

1. Subject demands to discontinue the study
2. Unusual changes in clinical test results
3. Principal investigator’s decision to terminate the study (low rates of compliance, complications, or inability to sustain the study for various reasons)

### Randomization

The first subject will be randomized to the control or intervention group by tossing a coin. Subsequently, the study subjects will be allocated alternatively to either group until such point that the expected sample number is reached. The subjects will be stratified according to sex, and an even quota of male and female subjects in both groups will be ensured. The allocation sequence will be closely monitored by two of the study authors (PF and RJ). Only the patients who satisfy the aforementioned inclusion and exclusion criteria will be selected for the study.

### Blinding

The patient management team, including the consultant cardiologist, medical officers, and nursing staff, will be blinded to the nature of the intervention to which the patient is assigned. All the interview-based questionnaires will be administered to the patients by a research assistant, who will make sure the patients of either group are blinded to the intervention or other group during questioning. During the clinic follow-up visits, the participants will be advised not to convey information regarding the nature of the intervention they are undergoing to other study participants or to the attending clinician to avoid cross-contamination or breach of the single-blind study design, respectively. Furthermore, the participants within the control and intervention groups will be followed on different working days to ensure the quality of the study.

### Intervention

#### Day 1

After the study protocol is explained by an investigator and after queries have been answered, written informed consent will be obtained from the individuals who satisfy the eligibility criteria. Once consent is obtained, the participants will be given an interviewer-administered questionnaire, which includes data on age, physical activity, area of residence, level of education, household income, coronary risk factors, diagnosis on admission, mode of management, and drugs given at the ward. Data on dietary habits will be assessed using a validated food frequency questionnaire (FFQ) [19] in an interviewer-administered format. Data on physical activity will be collected using the interviewer-administered International Physical Activity Questionnaire–Short Form (IPAQ-SF) [28]. Subjects in the “moderate”/“high” physical activity categories will be considered as being physically active.

Anthropometric measurements will be performed using calibrated equipment by a research assistant adhering to the WHO guidelines [29]. The exact methodology is described under the “Anthropometric measurements” subheading below.

#### Day 2 until discharge

Once the initial evaluation is over, fasting venous blood samples will be obtained by a trained nursing officer or medical graduate for glucose and lipid estimation from all participants in the ward setting. A blood sample will be taken after a 10-h fast. Plain tubes will be kept at room temperature for 30 minutes before separating serum. Once serum is separated, tubes will be separated into several aliquots for different investigations and stored at −20 °C by placing them in an icebox before transporting them to the laboratory.

During the hospital stay, the patients will be engaged in a rehabilitation program from day 2 onward, and it will be commenced in parallel with the study by a team trained by the study investigators. Patients will be provided with information about the disease, the basic anatomy of the heart, and a step-up exercise program that includes breathing and cardiovascular exercise, and further details will be provided to patients regarding general lifestyle modifications recommended in the post-MI period. The information is provided at a rate at which it can be absorbed by the patients, giving due regard to their psychological needs. Furthermore, aspects of healthy dietary habits will be addressed in detail to achieve the goals of the study. The checklist that will be used to evaluate the progression of the education sessions is given in Table 1.

**Table 1:** Post-myocardial infarction patient education checklist

Description		Date	✓
1	Basic structure of the heart		
2	What does heart attack mean?		
3	Treatment at the CCU		
4	What to do in case of a chest pain (i.e., use of GTN)		
5	Gradually increasing level of physical activity		
6	Personal care		
7	Return to work		
8	Driving		
9	Traveling		
10	Sexual activity		
11	Ways to minimize psychological stress		
12	Cessation of smoking		
13	Use of alcohol		
14	Diet		
15	Stress		
16	Sleep and rest		
17	Discharge drugs		
18	Emergency contacts, clinic follow-up visits		

*CCU* Coronary care unit, *GTN* Glyceryl trinitrate

As part of this 12-week hospital- and home-based intervention, each participant in the intervention group will be provided with a model plate along with the food poster and a food diary. The patients will also be provided with nutrition advice that will be customized according to patients’ social, economic, and personal preferences by a trained research assistant (PF) with close supervision of a nutrition specialist (RJ). Advice in other domains, such as physical activity and lifestyle modification (e.g., cessation of smoking, cutting down alcohol and intake of drugs), will be evenly provided to both the groups as part of routine care by the discharging nursing officer.

Two specially designed booklets will be provided to the patients to standardize the health education delivered to the patients verbally. The first booklet will contain information regarding the step-up plan of exercise following MI in both hospital and home settings. The second booklet gives information regarding lifestyle modifications following MI, such as cessation of smoking, regular sleeping, and resuming sexual activities. Both the booklets will be provided to the control and intervention groups. Furthermore, to enhance knowledge on different food choices under each category/color, a food poster will be given to the patients who will be following the plate model diet.

#### Follow-up visits

The adherence to the intervention among the study participants will be scrutinized with the use of a self-maintained food diary. The compliance will be reinforced by regular follow-up calls by a research assistant (PF) and monitored at follow-up clinic visits using the FFQ at 4 weeks and 12 weeks following recruitment of the study subject.

As part of the research, data will be gathered for qualitative assessment of the knowledge, perception, and attitudes of the patients regarding the plate model diet during the follow-up visit at 4 weeks. This qualitative research is an integral part of the study that will provide insight into aspects such as adherence, feasibility, and practical problems encountered when the plate model is used in the Sri Lankan context.

### Study groups

The intervention arm subjects will be provided with the model plate, whereas subjects in the control arm will be provided with routine care, which is the only difference between the two groups. Otherwise, the management and clinic follow-up visits will be continued according to routine for all the patients recruited into the study.

### Study materials

**Fig. 3:**
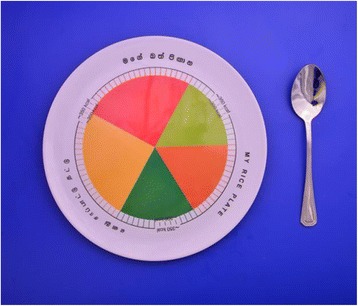
A photograph of “My Plate” that is provided to the patients

“My rice plate” is a 3D plate made up of melamine, and the diameter is 10.5 inches. The title of the plate is printed in both local languages (Sinhala and Tamil) and English over the outermost border of the plate (Fig. 3). The plate is divided into five segments by printed lines. One-quarter of the plate is for rice, and another quarter is for protein-containing foods such as meat, chicken, fish, eggs, and soya products (textured vegetable protein). The remaining half is for nonstarchy vegetables. The segment representing the vegetables is again divided into three equal areas: one-third for green leafy salads (*mellum* in traditional Sri Lankan terms) and the other two-thirds for two different vegetables, including one green vegetable (Fig. 3). Each segment is colored with a color specific to that particular food group.

The model plate will be supplemented with a food poster to educate patients on the food items in each group, which will also enhance compliance with the plate model diet. A food diary, which will be filled out by the patients while following the plate model diet, will be used to analyze compliance and will also be used as a monitoring tool during the follow-up visits. The model plate, food diary, and food poster will be the only intervention materials used in the study. A summary of interventions taking place for each group is provided in Table 2.

**Table 2:** Comparison of interventions between the two arms

Arm A: test group	Arm B: control group
1. Provision of model plate “My rice plate” is a 3D plate made of melamine, which is designed to control portion size of each food group with visual reinforcement with different color segments assigned for each food group. Food poster will be provided to improve compliance with plate model diet, and food diary will be used as a monitoring tool in follow-up visits. 2. Each patient is given one-to-one education, with each session lasting about 5–10 minutes per day until the discharge, which comprises information regarding exercise as well as general lifestyle modification in post-MI period, such as cessation of smoking and cutting down alcohol. 3. Two separate booklets will be provided to the patients free of charge to reinforce the health education delivered to the patients verbally. 4. The rest of the medical management will be provided on an individual basis according to the standard guidelines and ward protocols.	1. Routine dietary advice will be provided, such as reduced intake of fat, sugar, and salt. Interventions 2, 3, and 4 in the control group will be the same as for the test group.

*MI* Myocardial infarction

### Study period

The recruitment period is estimated to span 12 weeks. The follow-up period is estimated to be 12 weeks from the day of the last patient recruitment. A further 2 months will be spent on data finalization, analysis, and report writing. Therefore, a total period of 8 months is estimated as the study period.

### Outcomes

The primary outcome is mean body weight reduction of 10% in the test group following the plate model diet for a period of 12 weeks compared with the control group. The following secondary outcomes will be evaluated in the test group compared with the control group at the end of the completion of the 12-week period from commencement of intervention: reduction of systolic and diastolic blood pressure, BMI, waist circumference (WC), hip circumference, waist-to-hip ratio, FBS, serum total and LDL cholesterol, serum ALT, and total caloric intake/day (FFQ), as well as improvement in level of physical activity (based on IPAQ-SF) and mean increment in serum HDL cholesterol level.

### Study schedule

The detailed measurements that will be assessed at every visit are described in Table 3.

**Table 3:** A brief study schedule at every visit

	On admission	Visit 1 (1 month)	Visit 2 (3 months)
Informed consent form	*		
Recording demographic data	*		
Medical history taking	*		
Physical examination^a^	*	*	*
Blood pressure	*	*	*
FBS	*	*	*
ALT	*	*	*
Lipid profile	*		*
Drug chart	*	*	*
FFQ	*	*	*
IPAQ-SF	*	*	*

*Abbreviations: ALT* Alanine aminotransferase, *FBS* Fasting blood sugar, *FFQ* Food frequency questionnaire, *IPAQ-SF* International Physical Activity Questionnaire–Short Form

^a^ Body weight, height, waist circumference, and hip circumference

* performing given activity/investigation in that perticular visits

### Measurement tools

#### Anthropometric measurements

Anthropometric measurements will be performed using calibrated equipment by a research assistant adhering to the WHO guidelines [29]. Height will be taken to the nearest 0.1 cm, as the maximum distance to the uppermost position on the head from the heels, with the individual standing barefoot and in full inspiration using a standard stadiometer (Seca 823; Seca, Hamburg, Germany). Body weight will be measured to the nearest 0.1 kg using an electronic weighing scale (Seca 803) with the participants wearing light indoor clothing. BMI will be calculated as weight in kilograms divided by height squared in meters (kg/m^2^). WC will be measured midway between the iliac crest and the lower rib margin at the end of normal expiration using a plastic, nonelastic flexible tape to the nearest 0.1 cm. Hip circumference will be measured at the widest level over the greater trochanters using a plastic flexible tape to the nearest 0.1 cm. Seated BP will be measured after at least a 10-minute rest using a mercury sphygmomanometer (model 0125; Accoson, Harlow, UK).

#### Dietary measurements

A validated FFQ will be used as an interviewer-administered questionnaire by a trained research assistant to obtain habitual intake of calories as well as macro- and micronutrients. Data will be analyzed using NutriSurvey 2007 software (EBISpro Software, Willstätt-Legelshurst, Germany).

### Statistical analysis

Completed questionnaires will be checked before data entry. Data will be entered and analyzed using IBM SPSS Statistics version 21.0 statistical software (IBM, Armonk, NY, USA). One-sample Kolmogorov-Smirnov analysis will be used to test the distribution of variables. Characteristics of patients will be examined by chi-square tests for categorical variables and by Student’s *t* test and analysis of variance for continuous variables. Chi-square tests for independence will be used to compare weight changes between different BMI categories. Associations between biochemical parameters will be tested by Pearson’s correlation coefficient for normally distributed variables and Spearman’s correlation coefficient for variables that are not normally distributed. Statistical significance will be set at *p* < 0.05.

### Risks and benefits

The risks associated with the procedures will be negligible. The intervention is minor and expanded in a very mild manner compared with standard of care, but the expected benefits it would bring to individuals, the scientific community, and the health sector would be much higher. In case of any unlikely outcome such as unexpected weight loss or sudden deterioration of the disease condition of the patient, the intervention will be either discontinued or modified.

Investigation results will be sent to each subject under confidential cover. In the event that any abnormality is detected, subjects will be contacted by the investigators by phone and referred to relevant persons/institutes for further management according to their wish. Furthermore, for any clarifications during the study period, all the participants are provided with the contact information of the research assistant and the institute.

### Data collection

Data collection will be performed according to the hospital’s standard operating procedure by a research assistant.

### Monitoring and data management

Monitoring of data will be done on a weekly basis by the senior authors of the study (AP and RJ). All the data will be stored electronically in Excel spreadsheets (Microsoft, Redmond, WA, USA), and an SPSS database will be maintained and updated daily by a research assistant. Missing data and dropout will be stricken from the database and nullified. The sample size is calculated to compensate for missing data and dropouts throughout the study period.

### Security of data and samples

Data will be identified by a serial number, and except for the initial consent form, no biographical data will be collected. All hard copies of data will be kept under lock and key, and the soft data will be kept under password protection in the custody of the investigators and released for analysis and report formulation under supervision. Investigation reports transferred via the Internet will be accessible only to the investigators, and a password-protected portal system will be used to transfer such data.

All data will be destroyed 15 years after the final paper is published. All biological samples will be destroyed once data analysis is completed (in order to conduct duplicate analysis if required).

## Discussion

With accumulating knowledge on different strategies for coronary risk factor modification, the scientific evaluation of diet in regard to secondary prevention of IHDs is a necessity. In this article, we present a study protocol designed to evaluate the effects of the plate model as part of dietary intervention to modify cardiometabolic risk factors in post-MI patients over a period of 3 months. To the best of our knowledge, this will be the pioneering dietary interventional study in Sri Lanka and the South Asian region. Overall, this study would provide insight into the extent to which the plate model diet could be applied at the ground level in Sri Lanka. It is likely to uncover possible constraints that hinder the outcome of the results, such as economic barriers, myths and beliefs, lack of affordability, and unavailability of different foods. We believe that the plate model method is a simpler and more effective way of introducing meal planning that is an alternative to the traditional way of exchange-based teaching and meal planning. In this visual method, the plate serves as a pie chart depicting the proportions of the plate that should be covered by various foods. Camelon et al., who conducted the Diabetes Atherosclerosis Intervention Study, explained that this method could be practiced with patients to reinforce the connection between dietary theory and practice, promotion of memory retention, and use of a positive approach to nutrition counseling [30].

Acknowledging the limitations of the study, present studies show that using the IPAQ-SF may affect the outcome of the data because the correlation of the level of physical activity with objective methods of assessment was less than the acceptable standards [5]. Furthermore, the social desirability bias may affect the responses given to the FFQ, which is further confounded by the fact that patients with chronic diseases tend to receive a multitude of information from different sources on a daily basis, which may in turn affect their responses. Therefore, weight reduction over time has been chosen as the primary outcome, which is an objective measurement.

Finally, this study will provide insights into a practical model of dietary intervention that could be further developed by future studies to evaluate the long-term effects on morbidity and mortality of patients with CVD. The effectiveness of the model plate and its practicality in other populations with chronic diseases has to be evaluated.

### Trial status

Recruiting.

## Additional file

Additional file 1: SPIRIT 2013 checklist: recommended items to address in a clinical trial protocol and related documents. (DOC 122 kb)

## Abbreviations

ACS: Acute coronary syndrome
AHA: American Heart Association
ALT: Alanine aminotransferase
BMI: Body mass index
BP: Blood pressure
CCU: Coronary care unit
CVD: Cardiovascular disease
ECG: Electrocardiogram
FBS: Fasting blood sugar
FFQ: Food frequency questionnaire
GTN: Glyceryl trinitrate
HDL: High-density lipoprotein
IHD: Ischemic heart disease
IPAQ-SF: International Physical Activity Questionnaire–Short Form
LDL: Low-density lipoprotein
MI: Myocardial infarction
SPIRIT: Standard Protocol Items: Recommendations for Interventional Trials
STEMI: ST-elevation myocardial infarction
WC: Waist circumference
WHO: World Health Organization
